# Sleeping late is a risk factor for myopia development amongst school-aged children in China

**DOI:** 10.1038/s41598-020-74348-7

**Published:** 2020-10-14

**Authors:** Xiao Nicole Liu, Thomas John Naduvilath, Jingjing Wang, Shuyu Xiong, Xiangui He, Xun Xu, Padmaja R. Sankaridurg

**Affiliations:** 1 Brien Holden Vision Institute Limited, Sydney, Australia; 2 School of Optometry and Vision Science, University of New South Wales, Sydney, Australia; 3 Department of Preventative Ophthalmology, Shanghai Eye Disease Prevention and Treatment Center, Shanghai Eye Hospital, Shanghai, China; 4 Department of Ophthalmology, Shanghai General Hospital, Shanghai Jiao Tong University, Shanghai Key Laboratory of Ocular Fundus Diseases, National Clinical Research Center for Eye Diseases, Shanghai, China

**Keywords:** Patient education, Risk factors, Refractive errors, Paediatric research, Epidemiology

## Abstract

Myopia, a leading cause of distance vision impairment, is projected to affect half of the world’s population in 30 years. We analysed the relationship between certain demographic, environmental, and behavioural factors and myopia from a 2-year school-based, prospective trial conducted in Shanghai, China. This trial enrolled 6295 school-aged children at baseline and followed them up for 24 months. The relationship between abovementioned factors and myopia was examined and the role of sleep in childhood myopia development was highlighted. Our results suggest that ‘sleeping late’ is a risk factor for myopia prevalence at baseline (odds ratio [OR] = 1.55, *p* = 0.04), 2-year myopia incidence (odds ratio [OR] = 1.44, *p* = 0.02) and progression over 24 months (*p* = 0.005), after adjusting for residency area, age, gender, sleep duration, and time spent outdoors. The identification and consistency of results with late sleepers being a susceptible group to both myopia onset and progression suggests a complex relationship between circadian rhythm, indoor environment, habitual indoor activities and myopia development and progression. These results can offer new insights to future myopia aetiology studies as well as aid in decision-making of myopia prevention strategies.

## Introduction

Myopia, commonly known as ‘short-sightedness’ or ‘near-sightedness’, is predicted to affect approximately five billion people worldwide by 2050^1^. It is the most frequent cause of distance visual impairment in the world and results in enormous socio-economic burden^2,3^. Although there are many ways to correct the blurred distance vision caused by myopia, simple corrective strategies cannot halt the pathological changes that parallel continuous myopia progression. Such changes or complications can lead to irreversible visual impairment and, in severe cases, acquired blindness^4^. For example, myopic macular degeneration, one of the retinal abnormalities associated with myopia, has been identified as one of the leading causes of blindness and vision impairment in some parts of Asia^5–7^. Early onset of myopia in childhood will lead to extended courses of disease, which would not only result in a greater financial burden, but also produce higher risks for sight threatening complications^2^. Therefore, increase in the prevalence of myopia among children is particularly worrying.

Years of study has enabled the identification of a number of risk factors for myopia, such as family history of myopia, urban living environment, and a lack of outdoor exposure^8–10^. In recent years, a number of studies have investigated the relationship between myopia and sleep. Shorter sleep duration and poorer sleep quality was associated with greater myopic refractive error^11,12^. Altered light/dark or wake/sleep cycles were found to influence ocular growth patterns in animal studies^13,14^. Additionally, genetic factors regulating circadian rhythms were found to be involved in refractive error development in humans and retinal-specific knockouts of the clock gene were found to induce myopia in mice^15,16^. Furthermore, since the discovery of intrinsically photosensitive retinal ganglion cells (ipRGCs) in the retina, their association with melatonin and therefore sleep/wake cycle regulation, the role of sleep has become more intriguing for myopia research^17,18^. However, most myopia studies to date that investigated the role of sleep focused solely on the duration of it and there is a lack of longitudinal studies exploring the ‘timing’ or circadian rhythm aspect of sleep. Henceforth, we present our findings on both the duration and the pattern of sleep and their associations with childhood myopia from a 2-year prospective, school-based study.

## Method

### Study design

This trial (the Shanghai Time Outside to Reduce Myopia trial) was approved by the Shanghai General Hospital Ethics Committee and adhered to the tenets of the Declaration of Helsinki (ClinicalTrials.gov Identifier: NCT02980445). Detailed information on the study design has been published previously^19^. A total of 6295 children (2949 girls, 46.8%), aged between 6 to 9 years (7.2 ± 0.7 years) at baseline, from 24 schools across eight districts in Shanghai, were enrolled. This was a school-based prospective trial, where schools were randomised to either the control group or the test group with different amount of outdoor activities as an intervention. Written informed consent was obtained from parents/carers of children participating in the study. Parents/carers consented to providing information on their child’s activity via a mobile phone based questionnaire. Children with systemic or ocular pathology or used any myopia control treatment were identified via a screening procedure at baseline and excluded for data analysis. Cycloplegic auto-refraction was performed at baseline and repeated annually. The cycloplegic agent employed in this procedure was 1% cyclopentolate (Cyclogyl; Alcon, Fort Worth, Texas, USA).

The questionnaire employed in this trial was administrated online via a mobile phone app to parents or carers at baseline and repeated four times per year (two times during school sessions and two times during holiday seasons). There were four sections in the questionnaire, containing questions on demographics (e.g. gender, date of birth, and name of school), family history of myopia, daily amount of various activities on a typical school day (e.g. how much time the child spent on outdoor activities), eye-using habits (e.g. reading distance) and myopia management experiences (if applicable). The questionnaire is included as "Appendix 1". This article focuses on environmental and behavioural factors, especially on those related to sleep patterns, to determine whether any relationship exists between sleep/wake-up time and myopia.

### Definitions and classifications

Based on cycloplegic auto-refraction (KR-8900, Topcon, Tokyo, Japan) results, spherical equivalent (SE) was computed as sphere plus half cylinder. Myopia was defined SE ≤  − 0.5 dioptres (D); hyperopia was defined as SE ≥  + 0.75D; emmetropia was defined as SE >  − 0.50D and <  + 0.75D. Only right eyes were used for classifying refractive error. Incidence was defined as new myopic cases over 24 months among those who were not myopic at baseline. Progression was computed as a difference between 24-month and baseline SE.

For sleep pattern related questions, parents/carers were asked to recall and write down the specific time (in ‘hh:mm’ format) when the child went to sleep in the night and woke up in the morning on a usual school day.

### Statistical analysis

Outcome variables for analyses were myopia at baseline, myopia incidence and progression by the 24-month visit. Questionnaire data from baseline, 12 and 24 months were used for the analysis. The baseline model used questionnaire data from the baseline visit. The incidence model used questionnaire data from 12 and 24 months visit. Progression model at 24 month used questionnaire data from 24 month visit. Associations of various demographics, environmental and behavioural factors with outcome variables were investigated. Chi-square and t-test were used for univariate analysis. Factors that were significant at the univariate level were included for multivariate testing. Age, gender, and area (urban/suburban) were included in all multivariate models as possible confounders. In addition, as participants of this trial were randomly assigned to three groups (a control group and two test groups with varying amount of outdoor time per day), the potential impact of study groups were also accounted for in multivariate models for incidence and progression. Myopia prevalence at baseline and incidence over 24 months were analysed using multiple logistic regression, while 24 month progression from baseline was analysed using general linear model. Backward elimination followed by forward entry was used as a method of model development. The final model included only factors that were significant at the 5% level of significance. Cluster based robust estimate of variance was employed for the incidence model that used 12 and 24 month visits. Interactions of main effects were assessed using Likelihood Ratio test and if significant, was further explored using subgroups of the interacting factor. All analyses were performed using Stata/IC 15.1 (StataCorp LLC, Texas 77845 USA).

## Results

### Study sample characteristics

At baseline, data were available for 6042 participants (mean age 7.36 ± 0.60 years; female 2835, 46.93%), amongst which there were 409 cases of myopia and the prevalence was 6.77% (409/6024). Detailed baseline sample descriptive data can be found in a previous publication^19^. After 2 years, at the 24-month visit, data were available for 5355 participants and myopes accounted for 27.64% (1480/5355) of the study sample. A total of 4982 children who were not myopic at baseline attended the 24-month visit, amongst them 1094 became myopic during this 24-months time period. The 2-year incidence of myopia was 21.92% (1094/4982).

### Distribution of sleep-related variables

**Figure 1:**
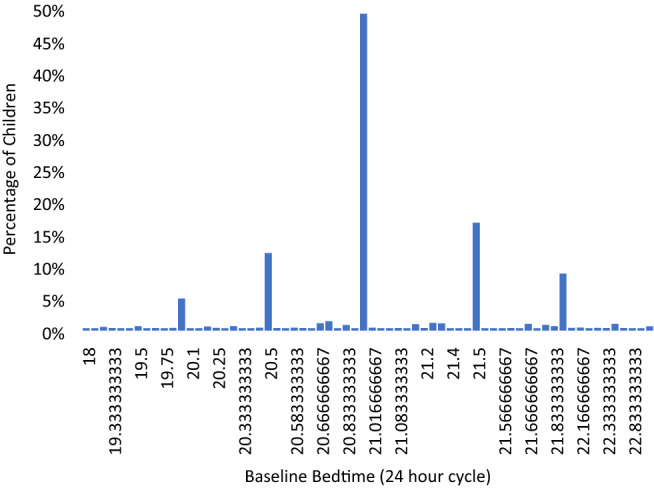
Distribution of bedtime at baseline.

**Figure 2:**
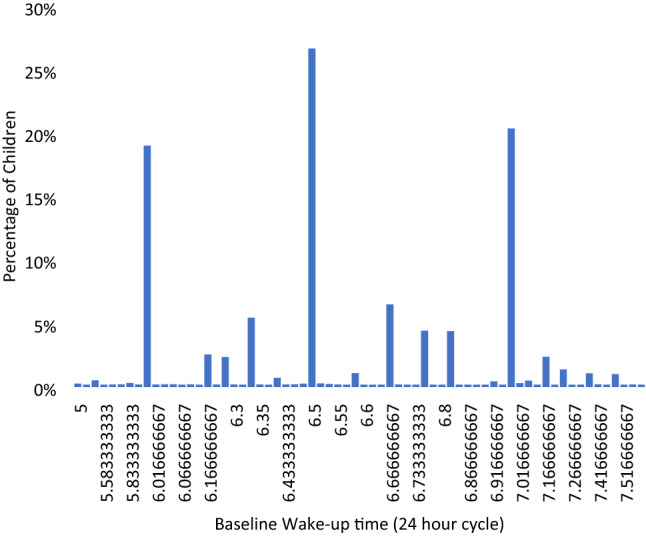
Distribution of wake-up time at baseline.

Prior to risk factor analyses, distributions of bedtime (time to sleep) and wake-up time of the cohort were examined. As shown in Figs. 1 and 2, distributions of both variables were not continuous but were clustered into distinct groups.

**Figure 3:**
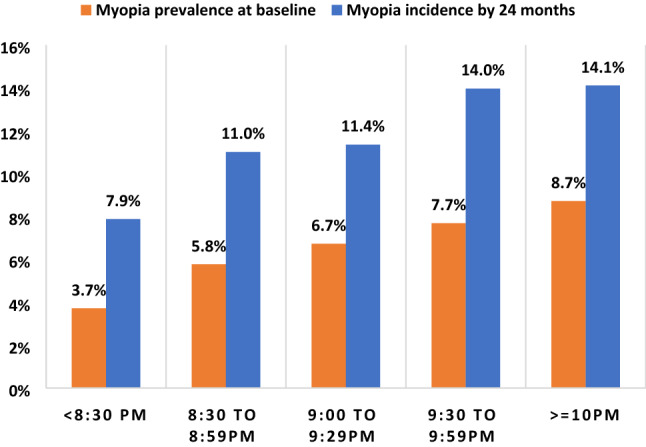
Trend of myopia baseline prevalence and 24-month incidence by bedtime categories.

When bedtime was categorised into groups based on the observed distribution (Fig. 1), bedtime was observed to be linearly associated with baseline myopia prevalence as well as incidence at 24-months (Fig. 3). Therefore, subsequent analyses considered bedtime and wake-up time as categorical data.

**Figure 4:**
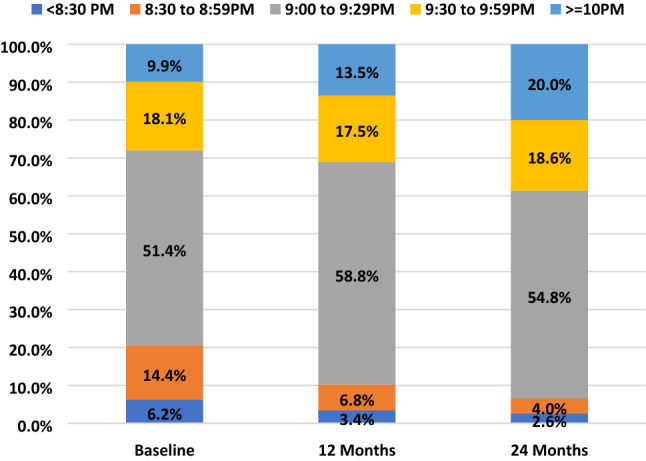
Percentage of children by bedtime at baseline, 12-month and 24-month visits.

We studied the sleep pattern of these children and found the average bedtime delayed from 21:04 at baseline to 21:17 at 24 months (One-way ANOVA, *p* < 0.001). Additionally, only 9.9% of children slept late (at 10 p.m. or after) at baseline, which, surprisingly, doubled to 20% by 24 months (Fig. 4). In contrast, the proportion of children who slept early (before 9 p.m.) decreased from 20.6 to 6.6% over this time period (Chi-square, *p* < 0.001).

**Figure 5:**
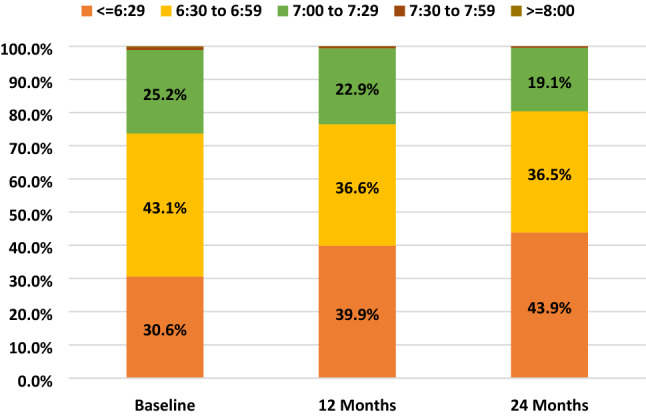
Percentage of children by wake-up time at baseline, 12-month and 24-month visits.

Meanwhile, the average wake-up time of children advanced over time from 6:34 at baseline to 6:27 at 24 months (One-way ANOVA, *p* < 0.001). The percentage of children who woke up early (before 6:30) also increased by 1.47 times (Fig. 5, Chi-square, *p* < 0.001). As a result, their average sleep duration decreased from 9.49 to 9.17 h over this time period (One-way ANOVA, *p* < 0.001).

### Sleep and baseline myopia

We compared myopes and non-myopes at baseline and investigated which demographic and/or behavioural factors were associated with myopia presented at baseline (Table 1). The univariate analysis shows that, apart from previously known risk factors, such as age, urban residency, family history of myopia and less outdoor activities, late bedtime (*p* = 0.009) and late wake-up time (*p* = 0.001) are also associated with baseline myopia.

**Table 1:** Relationships between myopia at baseline and other variables.

Variables	Category	Non-myope		Myope		Unadjusted OR (95% CI)	*p*-value
		N	% (row)	N	%(row)		
Residency area	Sub-urban	4537	93.93%	293	6.07%	1 (Reference)	
	Urban	1096	90.43%	116	9.57%	1.64 (1.31, 2.05)	*p* < 0.001
Age (years)	6	1874	97.10%	56	2.90%	1 (Reference)	
	7	2747	92.09%	236	7.91%	2.87 (2.14, 3.87)	*p* < 0.001
	8	1012	89.64%	117	10.36%	3.87 (2.79, 5.37)	*p* < 0.001
Gender	Male	2964	92.45%	242	7.55%	1 (Reference)	
	Female	2668	94.11%	167	5.89%	0.77 (0.63, 0.94)	*p* = 0.01
	0	2443	96.29%	94	3.71%	1 (Reference)	
Myopic parent	1	1883	91.90%	166	8.10%	2.29 (1.77, 2.97)	*p* < 0.001
	2	1025	88.59%	132	11.41%	3.35 (2.54, 4.40)	*p* < 0.001
Parent education level	Lower than undergrad	3481	94.00%	222	6.00%	1 (Reference)	
	Undergrad	1855	92.15%	158	7.85%	1.34 (1.08, 1.65)	*p* = 0.007
	Postgrad	225	90.36%	24	9.64%	1.67 (1.07, 2.60)	*p* = 0.02
Usual wake-up time	Before 6:30 a.m.	1749	94.54%	101	5.46%	1 (Reference)	*p* = 0.24
	6:30 to 6:59 a.m.	2437	93.69%	164	6.31%	1.17 (0.90, 1.50)	
	7 a.m. and after	1445	90.94%	144	9.06%	1.73 (1.33, 2.25)	*p* < 0.001
Usual bedtime	Before 9 p.m.	1178	94.85%	64	5.15%	1 (Reference)	
	9 to 9:29 p.m.	2894	93.26%	209	6.74%	1.33 (1.00, 1.77)	*p* = 0.05
	9:30 p.m. and after	1552	91.94%	136	8.06%	1.61 (1.19, 2.19)	*p* = 0.002
Sleep duration (h)	< 9.5	2315	92.90%	177	7.10%	1 (Reference)	
	≥ 9.5 and < 10	1846	93.37%	131	6.63%	0.93 (0.71, 1.17)	*p* = 0.53
	≥ 10	1463	93.54%	101	6.46%	0.90 (0.70, 1.16)	*p* = 0.43
Reading time weekly total (h)		16.25 ± 6.91		16.51 ± 7.43		1.01 (0.99, 1.02)	*p* = 0.45
Screen time weekly total (h)		9.49 ± 0.54		9.47 ± 0.51		0.98 (0.95, 1.01)	*p* = 0.16
Outdoor time weekly total (h)		7.26 ± 3.86		6.63 ± 3.83		0.96 (0.93, 0.98)	*p* = 0.002

When univariate significant factors were added into the multivariate model, the results (Table 2) show that sleeping late (late bedtime) is associated with higher odds of being myopic. The modelling steps indicated that sleeping late was a better predictor than waking up late. Although insignificant, sleep duration was retained in the model to ensure that the significance of bedtime was not affected by this factor. Even after taking sleep duration and weekly outdoor time into consideration, those who slept at 9:30 p.m. or later had 1.55-fold higher odds of being myopic at baseline compared to those who slept before 9 p.m. (*p* = 0.04). There was no significant interaction of bedtime with other main effects in the model (*p* > 0.20).

**Table 2:** Multivariate logistic regression model for bedtime and baseline myopia.

Variable	Adjusted OR (95% CI)	Test statistic, *p*
**Area: sub-urban**	1 (Reference)	
Urban	1.44 (1.12, 1.84)	z = 2.85, *p* = 0.004
**Age (years): 6 years**	1 (Reference)	
7 years	3.02 (2.21, 4.14)	z = 6.91, *p* < 0.001
8 years	4.08 (2.89, 5.75)	z = 8.00, *p* < 0.001
**Gender: Male**	1 (Reference)	
Female	0.77 (0.62, 0.95)	z = − 2.42, *p* = 0.02
**Usual bedtime: Before 9 p.m.**	1 (Reference)	
9 to 9:29 p.m.	1.38 (0.99, 1.94)	z = 1.88, *p* = 0.06
9:30 p.m. and after	1.55 (1.02, 2.34)	z = 2.06, *p* = 0.04
**Sleep duration (h): < 9.5 h**	1 (Reference)	
9.5 to 10 h	1.07 (0.83, 1.39)	z = 0.53, *p* = 0.60
10 h and more	1.23 (0.88, 1.72)	z = 1.22, *p* = 0.22
Outdoor weekly total (per h)	0.96 (0.93, 0.98)	z = − 3.13, *p* = 0.002

### Sleep and myopia incidence

Using a similar approach, we then examined the relationship between sleep and 2-year myopia incidence. Table 3 presents the association between each variable and new myopic cases that developed during this follow-up period. To sum up, sleeping late and waking up late, along with known risk factors, namely urban living environment, older age, family history of myopia and less outdoor time were found to be significantly associated with newly developed myopia over 24 months.

**Table 3:** Relationships between 2-year myopia incidence and other variables.

Variables	Category	Non-myope		New myope		Unadjusted OR (95% CI)	*p*-value
		N	% (row)	N	% (row)		
Residency area	Sub-urban	6619	88.02%	901	11.98%	1 (Reference)	
	Urban	1519	84.62%	276	15.38%	1.34 (1.14, 1.56)	*p* < 0.001
Age (years)	6	2775	89.46%	327	10.54%	1 (Reference)	
	7	3931	86.51%	613	13.49%	1.32 (1.14, 1.54)	*p* < 0.001
	8	1432	85.80%	237	14.20%	1.40 (1.16, 1.70)	*p* < 0.001
Gender	Male	4345	88.40%	570	11.60%	1 (Reference)	
	Female	3791	86.20%	607	13.80%	1.22 (1.07, 1.39)	*p* = 0.003
Parental myopia	0	3815	91.03%	376	8.97%	1 (Reference)	
	1	2688	86.63%	415	13.37%	1.57 (1.34, 1.83)	*p* < 0.001
	2	1213	78.16%	339	21.84%	2.84 (2.38, 3.37)	*p* < 0.001
Parent education	Lower than undergrad	5231	88.92%	652	11.08%	1 (Reference)	
	Undergrad	2500	84.80%	448	15.20%	1.44 (1.25, 1.65)	*p* < 0.001
	Postgrad	289	82.81%	60	17.19%	1.67 (1.21, 2.30)	*p* = 0.002
Usual wake-up time	Before 6:30 a.m.	3570	88.41%	468	11.59%	1 (Reference)	
	6:30 to 6:59 a.m.	2909	86.73%	445	13.27%	1.17 (1.01, 1.35)	*p* = 0.03
	7 a.m. and after	1656	86.25%	264	13.75%	1.22 (1.03, 1.44)	*p* = 0.02
Usual bedtime	Before 9 p.m.	737	89.99%	82	10.01%	1 (Reference)	
	9 to 9:29 p.m.	4712	88.19%	631	11.81%	1.20 (0.94, 1.54)	*p* = 0.14
	9:30 p.m. and after	2684	85.26%	464	14.74%	1.55 (1.21, 2.00)	*p* = 0.001
Sleep duration (h)	< 9.5	5023	87.02%	749	12.98%	1 (Reference)	
	≥ 9.5 and < 10	2028	87.64%	286	12.36%	0.95 (0.82, 1.10)	*p* = 0.46
	≥ 10	1080	88.38%	142	11.62%	0.88 (0.73, 1.07)	*p* = 0.20
Reading time weekly total		16.58 ± 7.33		17.10 ± 7.47		1.01 (1.00, 1.02)	*p* = 0.03
Screen time weekly total		6.77 ± 3.74		6.35 ± 3.74		0.97 (0.95, 0.99)	*p* = 0.001
Outdoor time weekly total		8.45 ± 4.12		7.89 ± 4.01		0.97 (0.95, 0.98)	*p* < 0.001

After adjusting for residency area, age, and gender, children who slept at 9:30 p.m. or later had 1.4-fold higher odds of developing myopia (*p* = 0.02), compared to those slept early (before 9 p.m.) (Table 4). Similar to the baseline myopia model, time to sleep (i.e. bedtime) took precedence over time to wake up (i.e. wake-up time) in the multivariate model. Outdoor time is identified as a protective factor against myopia onset (OR = 0.97, *p* < 0.001) while longer sleep duration is not significant in the myopia onset model (*p* = 0.93). The interaction of bedtime with other main effects of the model was not significant (*p* > 0.26) except for residency area (*p* = 0.003), where the association of late bedtime with newly developed myopia over 24 months was significant in the sub-urban area, but was not significant in the urban area (*p* = 0.63), which comprised 20% of the study sample.

**Table 4:** Multivariate logistic regression for usual bedtime and 2-year myopia incidence.

Variable	Adjusted OR (95% CI)	Test statistic, *p*
**Area: Sub-urban**	1 (Reference)	
Urban	1.25 (1.06, 1.48)	z = 2.64, *p* = 0.008
**Age (years): 6 years**	1 (Reference)	
7 years	1.33 (1.14, 1.55)	z = 3.59, *p* < 0.001
8 years	1.40 (1.15, 1.70)	z = 3.4, *p* = 0.001
**Gender: Male**	1 (Reference)	
Female	1.23 (1.08, 1.41)	z = 3.07, *p* = 0.002
**Usual bedtime: before 9 p.m.**	1 (Reference)	
9 p.m. to 9:29 p.m.	1.21 (0.91, 1.61)	z = 1.29, *p* = 0.20
9:30 p.m. and after	1.45 (1.05, 2.00)	z = 2.27, *p* = 0.02
**Sleep duration (h): < 9.5 h**	1 (Reference)	
9.5 to 10 h	1.02 (0.88, 1.20)	z = 0.3, *p* = 0.76
10 h and more	1.04 (0.82, 1.32)	z = 0.32, *p* = 0.75
Outdoor weekly total (per h)	0.97 (0.95, 0.99)	z = − 3.66, *p* < 0.001

*Odds ratios were adjusted for outdoor intervention.

### Sleep and refractive error progression

At the 24-month visit, the mean myopic progression of 5305 participants over 2 years was − 0.92 ± 0.77 D for an average baseline age of 7.4 ± 0.6 years (range 6 to 9 years). Over 2 years 3.93%, 2.61%, and 1.50% of the children progressed by 1D, 1.5D, and 2D respectively. Univariate regression reveals significant associations between urban area, female gender, parental myopia, higher level parental education, late wake-up time, late bedtime, more reading, less screen time, and less outdoor time and more myopic progression (Table 5).

**Table 5:** Relationships between 2-year myopic progression and potential variables.

Variables	Category	Sample	24-months Progression (± SD)	Beta Coef. (SE)	*p*-value
Residency area	Sub-urban	4218	− 0.89 ± 0.76	1 (Reference)	
	Urban	1087	− 1.03 ± 0.79	− 0.14 (0.03)	*p* < 0.001
Age (year)	6	1663	− 0.91 ± 0.78	1 (Reference)	
	7	2640	− 0.91 ± 0.77	0.001 (0.02)	*p* = 0.98
	8	1002	− 0.93 ± 0.76	− 0.02 (0.03)	*p* = 0.50
Gender	Male	2818	− 0.84 ± 0.75	1 (Reference)	
	Female	2486	− 1.00 ± 0.79	− 0.15 (0.02)	*p* < 0.001
Parental myopia	0	2268	− 0.79 ± 0.70	1 (Reference)	
	1	1798	− 0.94 ± 0.79	− 0.15 (0.02)	*p* < 0.001
	2	980	− 1.20 ± 0.83	− 0.41 (0.03)	*p* < 0.001
Parent education	Lower than undergrad	3321	− 0.85 ± 0.76	1 (Reference)	
	Undergrad	1710	− 1.04 ± 0.78	− 0.19 (0.02)	*p* < 0.001
	Postgrad	196	− 1.05 ± 0.80	− 0.21 (0.06)	*p* < 0.001
Usual wake-up time	Before 6:30 a.m.	2362	− 0.85 ± 0.75	1 (Reference)	
	6:30 to 6:59 a.m.	1900	− 0.94 ± 0.78	− 0.09 (0.02)	*p* < 0.001
	7 a.m. and after	1039	− 1.01 ± 0.80	− 0.12 (0.03)	*p* < 0.001
Usual bedtime	Before 9 p.m.	359	− 0.85 ± 0.81	1 (Reference)	
	9 to 9:29 p.m.	2918	− 0.87 ± 0.76	− 0.02 (0.04)	*p* = 0.58
	9:30 p.m. and after	2027	− 0.98 ± 0.78	− 0.13 (0.04)	*p* = 0.002
Sleep duration (h)	< 9.5	3578	− 0.92 ± 0.77	1 (Reference)	
	≥ 9.5 and < 10	1167	− 0.90 ± 0.77	0.02 (0.03)	*p* = 0.55
	≥ 10	556	− 0.93 ± 0.79	− 0.01 (0.04)	*p* = 0.76
Reading time weekly total in h		5656		− 0.005 (0.001)	*p* < 0.001
Screen time weekly total in h		5512		0.02 (0.003)	*p* < 0.001
Outdoor time weekly total in h		5559		0.01 (0.003)	*p* < 0.001

After adjusting for residency area, age, and gender, the multivariate general linear model in Table 6 presents strong evidence supporting the association of late bedtime with higher myopic progression (*p* < 0.001). Even after accounting for the effect of sleep duration and time spent outdoors, sleeping late was still a significant risk factor. Myopia progression in children who slept after 9:30 p.m. was 0.16 D greater on average compared to those who slept before 9 p.m., with the magnitude of progression increasing with later bedtime. Though one of the levels of sleep duration (> 10 h) appeared to have a detrimental effect, the multivariate model showed that the overall effect of longer sleep duration was not a significant factor (*p* = 0.13). Similarly, it was not significant in the univariate analysis (*p* > 0.5). There was no significant interaction of bedtime with other main effects in the model (*p* > 0.13).

**Table 6:** Multivariate general linear model for usual bedtime and myopic progression over 24 months.

Variable	Coef. (95% CI)	*p*-value
**Area: Sub-urban**	1 (Reference)	
Urban	− 0.11 (− 0.17, − 0.06)	*p* < 0.001
**Age (years): 6 years**	1 (Reference)	
7 years	− 0.003 (− 0.05, −0.04)	*p* = 0.91
8 years	− 0.02 (− 0.08, − 0.04)	*p* = 0.46
**Gender: Male**	1 (Reference)	
Female	− 0.15 (− 0.19, − 0.11)	*p* < 0.001
**Usual bedtime: Before 9 p.m.**	1 (Reference)	
9 p.m. to 9:29 p.m.	− 0.06 (− 0.16, − 0.03)	*p* = 0.21
9:30 pm and after	− 0.16 (− 0.26, − 0.05)	*p* = 0.004
**Sleep duration (hours): < 9.5 h**	1 (Reference)	
9.5 to 10 h	− 0.02 (− 0.08, − 0.03)	*p* = 0.41
10 h and more	− 0.09 (− 0.17, − 0.02)	*p* = 0.045
Outdoor weekly total (per h)	0.01 (0.004, 0.01)	*p* < 0.001

*Odds ratios were adjusted for outdoor intervention.

### Sleep pattern and risk factors for late bedtime

In an effort to understand why some children were late sleepers, we then explored factors associated with sleeping late in this cohort. Remarkably, the known risk factors for myopia were also significantly associated with late bedtime. Results in Table 7 show that sleeping late is more prevalent in children who: reside in urban areas (*p* < 0.001); are older aged (*p* = 0.01 and *p* = 0.04); have myopic parents (*p* < 0.001); have higher parental education level (*p* < 0.001); wake up late (*p* < 0.001); spent more time on reading (*p* < 0.001) and on screen (*p* = 0.03) per week; and/or spend less time outdoors per week (*p* = 0.002).

**Table 7:** Relationships between baseline usual bedtime and potential variables.

Variables	Category	Slept before 9:30 p.m.		Slept at 9:30 p.m. and after		Unadjusted OR (95% CI)	*p*-value
		N	% (row)	N	% (row)		
Residency area	Sub-urban	3657	75.86%	1164	24.14%	1 (Reference)	
	Urban	688	56.77%	524	43.23%	2.39	*p* < 0.001
Age (years)	6	1432	74.31%	495	25.69%	1 (Reference)	
	7	2114	70.96%	865	29.04%	1.18 (1.04, 1.35)	*p* = 0.01
	8	799	70.90%	328	29.10%	1.19 (1.01, 1.40)	*p* = 0.04
Gender	Male	2311	72.17%	891	27.83%	1 (Reference)	
	Female	2034	71.87%	796	28.13%	1.02 (0.91, 1.14)	*p* = 0.80
Myopic parent	0	1927	75.99%	609	24.01%	1 (Reference)	
	1	1428	69.90%	615	30.10%	1.36 (1.20, 1.56)	*p* < 0.001
	2	769	66.46%	388	33.54%	1.60 (1.37, 1.86)	*p* < 0.001
Parent education	Lower than undergrad	2823	76.36%	874	23.64%	1 (Reference)	
	Undergrad	1322	65.77%	688	34.23%	1.68 (1.49, 1.89)	*p* < 0.001
	Postgrad	140	56.22%	109	43.78%	2.51 (1.94, 3.27)	*p* < 0.001
Usual wake-up time Sleep duration (hours)	Before 6:30 a.m.	1657	89.62%	192	10.38%	1 (Reference)	
	6:30 to 6:59 a.m.	1862	71.70%	735	28.30%	3.41 (2.87, 4.05)	*p* < 0.001
	7 a.m. and after	826	52.05%	761	47.95%	7.95 (6.65, 9.51)	*p* < 0.001
	< 9.5	1215	48.76%	1277	51.24%	1 (Reference)	
	≥ 9.5 and < 10	1587	80.27%	390	19.73%	0.23 (0.20, 0.27)	*p* < 0.001
	≥ 10 h	1543	98.66%	21	1.34%	0.01 (0.01, 0.02)	*p* < 0.001
Reading time weekly total		15.93 ± 6.79		17.14 ± 7.24		1.03 (1.02, 1.03)	*p* < 0.001
Screen time weekly total		6.70 ± 3.80		6.94 ± 4.02		1.02 (1.00, 1.03)	*p* = 0.03
Outdoor time weekly total		7.32 ± 3.85		6.97 ± 3.87		0.98 (0.96, 0.99)	*p* = 0.002

When comparing sleep patterns between weekdays and weekends, children were found to wake up by almost one hour later during weekends than during weekdays (mean difference − 0.80 h, 95% CI [− 0.82, − 0.77], *p* < 0.001) whereas they went to sleep at a similar time as week days (mean difference − 0.25 h, 95% CI [− 0.26, − 0.24], *p* < 0.001). This suggests that the time that the child went to sleep (i.e. bedtime) may be a more robust variable for sleep patterns in children, compared to wake-up time.

## Discussion

Our results suggest that, in this age group of 6 to 10 years, sleeping late (after 9:30 p.m.) significantly increased the risk of myopia. Late bedtime was seen to be a consistent risk factor associated with higher prevalence of myopia at baseline, higher incidence of myopia over 2 years, and greater progression of refractive error over 2 years.

Although the current study sample is much larger, younger, and without high degrees of myopia, our findings do echo with a previous study published by Ayaki et al., where a group of 21 highly myopic teenagers (mean age 16.7 ± 2.4 years) were found to have a late bedtime compared to teenagers with either mild or no myopia^12^. The authors also found this group had the shortest sleep duration. In our study, sleep duration did not differ between myopes and non-myopes at baseline (9.49 vs. 9.47 h, *p* = 0.6) or at 24-month visit (9.16 vs. 9.18 h, *p* = 0.6). While our findings did not reveal any relationship between sleep duration and myopia, a number of previous studies have reported significant yet contradictory results. Some studies found sleeping less promotes myopia, whilst others found the opposite. For example, one study involving 3625 Korean teenagers (age range 12–19 years) identified an inverse relationship between sleep duration and the severity of myopia^11^. Subjects who had more than nine hours of sleep were 41% less likely to have myopia compared to those who slept less than 5 h per night, after adjusting for myopia related risk factors. Similarly, results from a study that enrolled 15,136 Chinese children (age range 6 to 18 years) indicated an increased risk for myopia (adjusted-OR = 3.37) amongst those who slept less than 7 h per night compared to those who slept more than 9 h per night^20^. In contrast, another study of 1902 Chinese children (mean age 9.80 ± 0.44 years) identified a higher risk for myopia amongst those who slept longer every night (OR = 1.02, 95% CI [1.01, 1.04]), after adjusting for age, gender, sleep disorder score, weekly near work and outdoor hours^21^. As previously iterated, our study demonstrated no evidence supporting a relationship between sleep duration and myopia, which is similar to the conclusion drawn by a Singaporean study on 376 infants, where they found no association between sleep duration at 12 months and myopia at 3 years^22^. A recent Chinese study also reported negative results for sleep duration and myopia progression, although the association became significant in girls after stratifying the sample by gender^23^. Moreover, unlike the Korean adolescent sample studied by Jee et al. or the Chinese children sample studied by Xu et al.^11,20^, children of the current sample had sufficient sleep for their age group (an average of 9.49 ± 0.54 h per night at baseline) according to consensus recommendations developed by the American Academy of Sleep Medicine, who recommends at least 9 h sleep every night for 6 to 9 years old^24^. This, perhaps, along with the relatively narrow age range of our sample (6 to 9 years compared to up to 19 years in other studies), are the reasons why sleep duration did not stand out as a significant factor here.

Our findings highlight the impact of sleeping late on myopia onset and progression, although the underlying mechanisms remain unclear. On the one hand, sleeping late could hint at more late-night myopigenic activities and more exposure to artificial lighting conditions of the child. In the evening, while staying in an indoor environment, a child is highly likely to spend more time on near-based activities, such as reading or on digital screens. The impact of excessive near work on myopia development has already been broadly studied^25–27^, although the ‘timing’ factor has never been discussed in those studies. This probably also contributed to the inconsistency findings on the relationship of near work and myopia^26,28,29^. The effect of artificial lighting is another frequently investigated yet still equivocal topic amongst myopia studies^30–35^. Due to inconsistent results seen in animal models and the multi-dimensional complexity of artificial lighting^34^, much more research is needed before optimal artificial lighting conditions can be identified, if at all, for myopia prevention. Additionally, children who read more and spent more time on screen and less time outdoors were found more likely to sleep late (Table 7), which echoes with previous discussion by Morgan et al. that increased education pressure is a risk factor for myopia^36^.

On the other hand, sleep-related ‘timing’ or ‘time-of-day’ variable, is in fact a circadian rhythm marker and interests have already been mounting around the relationship between circadian rhythms and myopia^16,18,37,38^. Sleep–wake cycle is perhaps the most frequently perceived example of circadian rhythms by human beings. As a consequence of involuntarily shifting between two ‘time zones’: one determined by our internal clock, the circadian rhythm, and the other governed by study, work or other social duties in modern societies, misalignment of biological and social time is almost universal^39^. Disturbance to the circadian clock can not only affect the academic performance of children^40^, but also cause several health problems^41–43^. In terms of the visual system, a number of animal studies have demonstrated the importance of regular rhythmicity of lighting conditions on normal ocular growth and emmetropisation^13,14,44^. For example, retinal-specific knockouts of the clock gene can induce myopia in mice^16^. In humans, circadian dysregulation has been reported for myopic subjects. Compared to non-myopes, myopes were found to have higher serum concentration of melatonin in the morning, shorter sleep duration, and poorer sleep quality^11,12,45^. Seasonal changes reported in myopia progression could also suggest an impact of seasonal variations of circadian rhythms on the development of myopia^46^. Evidence supporting a role of circadian rhythm in myopia development is further strengthened by the results from a recent meta-analysis of genome-wide association studies, where genetic factors regulating circadian rhythm are identified to also participate in the development of myopia and refractive error^15^. Finally, yet importantly, several ocular biometry parameters, such as intraocular pressure (IOP), choroidal thickness, and axial length, were found to exhibit diurnal rhythms in humans^47,48^. Nickla et al. identified in chicks a positive correlation between the phase difference in axial length and choroid thickness and ocular growth rate and proposed that such phase difference could be seen as a predictor for eye growth rate^49^. The author further suggested that myopia treatment could incorporate ‘timing’ as a factor in implementation in order to achieve the best possible outcome^37^.

Evidence for a complex relationship between circadian rhythm and myopia development is mounting, despite the fact that the underlying mechanisms are yet to be illuminated. Nevertheless, our results can confirm that sleeping late is closely associated with myopia, but additional research is needed to determine whether sleeping late makes children more prone to myopigenic activities under poor lighting conditions when they are supposed to be sleeping or more susceptible to abnormal eye growth due to circadian disturbance. These are just two of the many more questions for future myopia studies.

The strengths of the current study include the large sample size and a school-based design of the trial. The extensive support received from the local governmental and school personnel made it a logistically successful trial with good data collection. The mobile phone app-based questionnaire provided a convenient way to address the questionnaire and enabled timely data entry by the parents/carers, thus minimising data entry errors associated with transfer of data from paper-based questionnaires to digital forms. Furthermore, refractive error was determined by cycloplegic auto-refraction and therefore increasing the confidence in the refractive error data. Yet, there were a number of limitations. To begin with, data collected via questionnaires are subject to recall bias. Although a wearable device was used in this trial, it was not worn after 7 p.m. and therefore sleep-related information was not captured. Secondly, although the sample size was large, the trial sample was localised and derived from the metropolitan Shanghai with children sharing same ethnic and cultural background. This might make the findings of current study less generalisable to other populations. Finally, the age range of the current cohort was relatively narrow (6 to 9 years old at baseline). Therefore, further studies focusing on samples from other regions, wider age range and more diversity are desirable to determine if these results hold valid for the wider population.

## Conclusion

This study has shown that sleeping late is a risk factor for higher prevalence, higher incidence, and greater progression of myopia in urban Chinese primary school children. The association between late bedtime and childhood myopia development may allude to a more complex relationship of indoor environment, activities, circadian rhythm and myopia, which needs to be further explored.

## Supplementary information

Supplementary Appendix.

## Data Availability

The datasets generated during and/or analysed during the current study are available from the corresponding authors on reasonable request.
