# Publisher Correction: Sleeping late is a risk factor for myopia development amongst school-aged children in China

**DOI:** 10.1038/s41598-021-84377-5

**Published:** 2021-02-23

**Authors:** Xiao Nicole Liu, Thomas John Naduvilath, Jingjing Wang, Shuyu Xiong, Xiangui He, Xun Xu, Padmaja R. Sankaridurg

**Affiliations:** 1 Brien Holden Vision Institute Limited, Sydney, Australia; 2 School of Optometry and Vision Science, University of New South Wales, Sydney, Australia; 3 Department of Preventative Ophthalmology, Shanghai Eye Disease Prevention and Treatment Center, Shanghai Eye Hospital, Shanghai, China; 4 Department of Ophthalmology, Shanghai General Hospital, Shanghai Jiao Tong University, Shanghai Key Laboratory of Ocular Fundus Diseases, National Clinical Research Center for Eye Diseases, Shanghai, China

Correction to: *Scientific Reports* 10.1038/s41598-020-74348-7, published online 14 October 2020

This Article contains an error in Figures 1 and 2 where the decimal place values on the x-axes are incorrectly captured.

The correct Figures 1 and 2 appear below.

**Figure 1:**
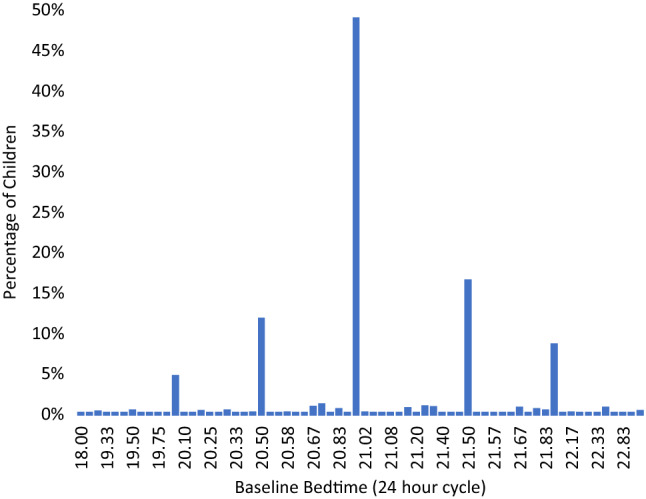
A correct version of the original Figure 1.

**Figure 2:**
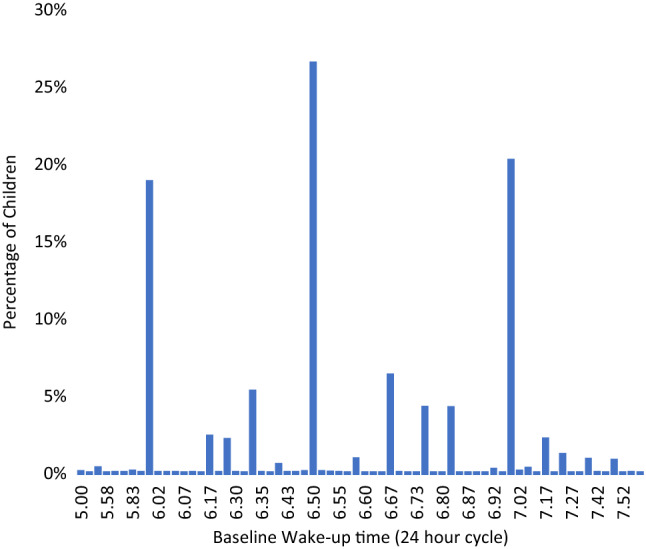
A correct version of the original Figure 2.

