# Supplementary material for: Sleeping late is a risk factor for myopia development amongst school-aged children in China
Source: Sci Rep. 2020 Oct 14;10:17194. doi: 10.1038/s41598-020-74348-7 (PMC7566837; doi:10.1038/s41598-020-74348-7)
Supplement: Supplementary file 1 — Supplementary Appendix. [file 41598_2020_74348_MOESM1_ESM.docx]

**Sleeping late is a risk factor for myopia development amongst school-aged children in China**

Xiao Nicole Liu*^1,2^, Thomas John Naduvilath^1,2^, Jingjing Wang^3^, Shuyu Xiong^4^, Xiangui He^3,4^, Xun Xu*^3,4^, Padmaja R. Sankaridurg*^1,2^.

1. Brien Holden Vision Institute Limited, Sydney, Australia.
2. School of Optometry and Vision Science, University of New South Wales, Sydney, Australia.
3. Department of Preventative Ophthalmology, Shanghai Eye Disease Prevention and Treatment Center, Shanghai Eye Hospital, Shanghai, China.
4. Department of Ophthalmology, Shanghai General Hospital, Shanghai Jiao Tong University, Shanghai Key Laboratory of Ocular Fundus Diseases, National Clinical Research Center for Eye Diseases, China.

Dear parents,

We’d like to invite you to complete the following questionnaire that aims to determine factors influencing children’s myopia. The results will aid Shanghai municipal government develop eye care strategies for children that promote eye health.

Thank you very much for your support and cooperation!

Shanghai Eye Disease Prevention and Treatment Center (Shanghai Eye Hospital)

Department of Ophthalmology, Shanghai General Hospital, Shanghai Jiao Tong University

Baoshan District Center for Disease Control and Prevention

2016.6

| **Question No.** | **Participant Details** | |
| --- | --- | --- |
| 0.1 | Participant ID number | Stick a label  (These parts of information will get from schools in advance.) |
| 0.2 | Name of child |  |
| 0.3 | Date of birth |  |
| 0.4 | Gender |  |
| 0.5 | Name of school child attends |  |
| 0.6 | Person completing this questionnaire | ○ Father  ○ Mother  ○ Child himself/herself  ○ Others, please specific: _____________ |
| 0.7 | Date complete | ______Y______M______D |

**Part 1 Basic information**

| **Question No.** | **Birth History** | | | | |
| --- | --- | --- | --- | --- | --- |
| 1.1 | At birth | ____________________________(weight in kg)  ___________________________ (length in cms) | | | |
| 1.2 | Was the child born | ① Full term (≥37 weeks)  ② Pre term (<37 weeks) | | | |
| **Information regarding the parents of the child** | | | | | |
|  | | **Father** | | **Mother** | |
| 1.3 | Date of birth | ______Y______M______D | | ______Y______M______D | |
| 1.4 | Educational level | ① Junior middle school equivalent or less  ②Senior middle school equivalent(Technical school)  ③ Undergraduate degree (Junior college/college)  ④ Postgraduate degree (Masters, PhD) | | ① Junior middle school equivalent or less  ②Senior middle school equivalent(Technical school)  ③ Undergraduate degree (Junior college/college)  ④ Postgraduate degree (Masters, PhD) | |
| 1.5 | Has myopia | ①Yes **(If Yes, please answer question 1.6 & 1.7 & 1.8)**  ② No **(If No, please answer question 1.8)**  ③ Don’t know **(If Don’t know, please answer question 1.8)** | | ①Yes **(If Yes, please answer question 1.6 & 1.7 & 1.8)**  ② No **(If No, please answer question 1.8)**  ③ Don’t know **(If Don’t know, please answer question 1.8)** | |
| **If yes to myopia** | | **Right eye** | **Left eye** | **Right eye** | **Left eye** |
| 1.6 | Degree of myopia | ①＜3.00D  ②3.00～6.00D  ③＞6.00D  ④Don’t know | ①＜3.00D  ②3.00～6.00D  ③＞6.00D  ④Don’t know | ①＜3.00D  ②3.00～6.00D  ③＞6.00D  ④Don’t know | ①＜3.00D  ②3.00～6.00D  ③＞6.00D  ④Don’t know |
| 1.7 | Age at first pair of glasses | ①<= 12 years old  ②13-15 years old  ③>= 16 years old  ④Don’t know | | ①<= 12 years old  ②13-15 years old  ③>= 16 years old  ④Don’t know | |
| 1.8 | Monthly income of family  (including all sources of income) | ①less than 2000 RMB ②＞2000 but ≤4000 RMB  ③＞4000 but ≤ 6000 RMB ④＞6000 but ≤ 8000 RMB  ⑤＞8000 but ≤ 10000 RMB ⑥＞10000 but ≤ 15000 RMB  ⑦＞15000 but ≤ 20000 RMB ⑧＞20000 but ≤ 50000 RMB  ⑨more than 50000 RMB | | | |

**Part 2: Thinking of what your child did yesterday or last school day**

**Firstly,**

What time did they wake up? _____:_____

What time did they go to bed? _____:_____

Time school started _____:_____

Time school finished _____:_____

How much time was spent travelling to and from school? _____ (hr) ____ (mins)

How did they travel to school (bus/car/motorcycle/walk)?

**Continue to think of what your child did yesterday or last school day. Now we will ask questions related to indoor time. Please ask your child or other members in family if you are unsure?**

Before school- how much time did they spend reading/writing/study? _____ (hr) ____ (mins)

Before school- how much time did they watch TV or play computer/phone games? _____ (hr) ____ (mins)

After school - how much time did they spend reading/writing/study? _____ (hr) ____ (mins)

After school- how much time did they watch TV or play computer/phone games? _____ (hr) ____ (mins)

**Continue to think of what your child did yesterday or last school day. Now we will ask questions related to outdoor time. Please ask your child or other members in family if you are unsure?**

Before school- how much time did they spend playing outside? _____ (hr) ____ (mins)

After school- how much time did they spend playing outside? _____ (hr) ____ (mins)

**Part 3 Eye-using habits:** Please recall your child’s eye-using behavior listed below in **the last seven days**, and choose according to the actual situation of the child or ask the child's true experience

| **Question No.** | **Questions** | **General situation in the last 7 days** | | | |
| --- | --- | --- | --- | --- | --- |
| **3.1a** | Does the child rest his / her eyes after a period of continuous reading? | ① Yes; ② No | | | |
| **3.1b** | **If “Yes”,** how long does he/she continuously read before a rest? | ①Less than 0.5h ②0.5~1h  ③1~1.5h ④1.5 ~ 2h ⑤More than 2h | | | |
| **3.2a** | Does the child rest his / her eyes after a period of continuous watching TV? | ① Yes; ② No | | | |
| **3.2b** | **If “Yes”**, how long does he/she continuously watch TV before a rest? | ①Less than 0.5h ②0.5~1h  ③1~1.5h ④1.5 ~ 2h ⑤More than 2h | | | |
| **3.3a** | Does the child rest his / her eyes after a period of continuous computer use? | ① Yes; ② No | | | |
| **3.3b** | **If “Yes”**, how long does he/she continuously use computer before a rest? | ①Less than 0.5h ②0.5~1h  ③1~1.5h ④1.5 ~ 2h ⑤More than 2h | | | |
| **3.4** | If he/she has a habit of resting eyes, which are the methods used for resting ther eyes? (Choose one ore more) | ①Overlooking to rest eyes ②Slightly move around to rest eyes  ③Look at long or short distance alternatively to rest eyes  ④Eye excersises to rest eyes ⑤Close eyes to rest  ⑥Outdoor activities to rest eyes ⑦Look at green plants to rest eyes | | | |
| **3.5** | What is the child’s common reading distance (from eyes to book)? | ①Less than 10cm | ②11-20cm | ③21-30cm | ④More than 30cm |
| **3.6** | What is the child’s common viewing distance while watching TV (Eyes to TV)? | ①Less than 1m | ②1-1.5m | ③1.5-2m | ④More than 2m |
| **3.7** | What is the child’s common computer usage distance (eyes to computer)? | ①Less than 15cm | ②15-30cm | ③31-50cm | ④More than 50cm |
| **3.8** | 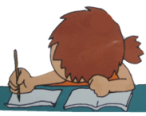Offset head when writing | ①never | ②Some times | ③often | ④always |
| **3.9** | 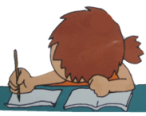Lie down on back when reading | ①never | ②Some times | ③often | ④always |
| **3.10** | Lie down on stomach when reading | ①never | ②Some times | ③often | ④always |
| **3.11** | Adequate light when reading and writing | ①never | ②Some times | ③often | ④always |

**Part4-1 Myopia and related treatments – for your child:** please tick accordingly.

| **Question No.** | **Questions** | **Choices** |
| --- | --- | --- |
| 4.1 | Does your child have myopia? | ①Yes  ②No **(The questionnaire is finished)**  ③Don’tknow **(The questionnaire is finished)** |
| 4.2 | At what age did your child develop myopia? | ______________years old or (or ____y____m_____d) |
| 4.3 | Please provide details when you first found your child develop myopia: | \|  \| Degree of myopia \| Degree of astigmatism \| \| --- \| --- \| --- \| \| Right eye \|  \|  \| \| Left eye \|  \|  \|   ○ Don’t know/Don’t remember |
| 4.5 | How often do you take your child to the hospital (or optical shop) for examination? | Every  ① 3 months or less  ② 6 months  ③ 1 year  ④ More than 1 year  ⑤ Not taken after initial exam.  ⑥ Cannot remember. |
| 4.6 | How did your child’s myopia progress in the last year? | The degree  ①Decreased.  ②Remained same.  ③Increased- less than 50 (not include 50 degrees).  ④Increased 50-100 (not include 100 degrees).  ⑤Increased more than 100 (include 100 degrees).  ⑥Cannot remember. |

**Part4-2 Does your child use or has used any of the following treatments for myopia?** Please choose according to the actual situation of your child and fill in the blanks.**（Multiple choices）**

| **Questions** | | **Treatments (Multiple choices)** | | | | |
| --- | --- | --- | --- | --- | --- | --- |
|  |  | **a. Frame spectacles** | **b. Ortho-K** | **c. Rigid Gas Permeable Contact lens (RGP)** | **d.Other methods**  **eg____________** | **e.Atropine** |
| 4.7 Does your child receive any of the listed myopia correction methods? | | ① Yes **(If Yes, please answer question 4.8-4.10 )**  ② No | ① Yes **(If Yes, please answer question 4.8-4.10 )**  ② No | ① Yes **(If Yes, please answer question 4.8-4.10 )**  ② No | ① Yes **(If Yes, please answer question 4.8-4.10 )**  ② No | ① Yes **(If Yes, please answer question 4.8-4.10 )**  ② No |
| **If Yes, please answer question 4.8 -4.10** | 4.8 The age start to wear(years) |  |  |  |  |  |
|  | 4.9 Up to now, is your child still receive any of the listed treatment? | ①Yes  ②No, please specific how long did you receive this treatment:  ________month. | ①Yes  ②No, please specific how long did you receive this treatment:  _______month. | ①Yes  ②No, please specific how long did you receive this treatment:  ________month. | ①Yes  ②No, please specific how long did you receive this treatment:  ________month. | ①Yes  ②No, please specific how long did you receive this treatment:  ________month. |
|  | 4.10 Frequency of changing or using? | ①every 6 months or more frequent  ②every year  ③every two years  ④every three years  ⑤Others,  please specific : ______  ⑥only have one pair, have not changed yet | ①every 6 months or more frequent  ②every year  ③every two years  ④every three years  ⑤Others,  please specific : ______  ⑥only have one pair, have not changed yet | ①every 6 months or more frequent  ②every year  ③every two years  ④every three years  ⑤Others,  please specific : ______  ⑥only have one pair, have not changed yet | ①every 6 months or more frequent  ②every year  ③every two years  ④every three years  ⑤Others,  please specific : ______  ⑥only have one pair, have not changed yet | ①Twice a week  ②Once a week  ③Every two weeks  ④Others,  please specific : ______ |

**It’s the end of the questionnaire, thanks again for your cooperation!**
